# Subjects Agree to Participate in Environmental Health Studies without Fully Comprehending the Associated Risk

**DOI:** 10.3390/ijerph8030830

**Published:** 2011-03-11

**Authors:** Robin Lee, Samantha Lampert, Lynn Wilder, Anne L. Sowell

**Affiliations:** Division of Health Studies, U.S. Agency for Toxic Substances and Disease Registry, 4770 Buford Highway NE, MS F-57, Atlanta, GA 30341, USA; E-Mails: HGZ4@cdc.gov (S.L.); LXW2@CDC.GOV (L.W.); ALS1@cdc.gov (A.L.S.)

**Keywords:** comprehension, consent forms, bioethics, environmental health, ethics, informed consent, research subjects

## Abstract

Recent advances in environmental health research have greatly improved our ability to measure and quantify how individuals are exposed. These advances, however, bring bioethical uncertainties and potential risks that individuals should be aware of before consenting to participate. This study assessed how well participants from two environmental health studies comprehended consent form material. After signing the consent form, participants were asked to complete a comprehension assessment tool. The tool measured whether participants could recognize or recall six elements of the consent form they had just reviewed. Additional data were collected to look for differences in comprehension by gender, age, race, and the time spent reading the original consent form. Seventy-three participants completed a comprehension assessment tool. Scores ranged from 1.91 to 6.00 (mean = 4.66); only three people had perfect comprehension scores. Among the least comprehended material were questions on study-related risks. Overall, 53% of participants were not aware of two or more study-related risks. As environmental public health studies pose uncertainties and potential risks, researchers need to do more to assess participants’ understanding before assuming that individuals have given their ‘informed’ consent.

## 1. Background

*Environmental Public Health Research.* While environmental public health research is not a new field, in recent years advances in technology have greatly improved our ability to measure and quantify how individuals are exposed. For example, biomonitoring and genetic research are two tools environmental health scientists are using more frequently as advances in these fields improve our ability to understand environmental influences on individuals and communities. Although these tools are revolutionary resources, there are new bioethical uncertainties, interpretative challenges, and potential risks that individuals who agree to participate in these studies should know [1–4].

*Informed Consent.* In conducting ethical research, scientists inform individuals about these risk factors via a consent process so that each individual can voluntarily decide for him or herself whether they want to participate. The process of obtaining informed consent implements safeguards designed to protect the welfare, privacy, and legal rights of study participants [5]. While obtaining information consent is ethically necessary a number of studies have found that participants have limited comprehension of the consent form materials they are given. Thus their decision to participate may not be based on the inherent risks and benefits of study participation. While this issue has been widely documented among specific subpopulations such as the elderly, substance abusers, the mentally challenged, or participants in clinical trials [6–13] To our knowledge, no studies have measured comprehension of consent material provided to the broader population involved in general environmental public health research. Therefore, this study measured the comprehension (using recognition and recall) of consent form material provided to individuals in one of two environmental health studies. The study also ascertained whether certain demographic factors (*i.e.*, gender, age, race) or the amount of time spent reviewing the form were associated with the ability to recognize or recall specific information.

## 2. Methods

*Study Population.* Comprehension of consent form material was measured among study participants from two environmental health studies conducted by the U.S. Agency for Toxic Substances and Disease Registry (ATSDR). The first study was an asbestos screening program (NAHP) and the second was a study on the variation in urinary creatinine and dissolved solids (VUCS). These two studies were selected to measure comprehension of consent form material because they were conducted by the same team of scientist at ATSDR and because both studies were implemented during a similar time period. Specific details on the consistency of the informed consent process within each study are further described below.

The purpose of the NAHP was to assess the development of radiological and pulmonary changes associated with exposure to asbestos-contaminated vermiculite. The target population included current and former workers from U.S. vermiculite processing facilities and their family members. Participants were offered a chest x-ray and spirometry test. Letters were used to introduce subjects to the study. The letter was followed-up by a telephone call. Both the letter and telephone call provided the subject with basic information about the study (e.g., study’s title, who was conducting the study).

The VUCS assessed variation in urinary creatinine and dissolved solid levels in children and young adults. The target population for the study included adults who currently worked for the public health agency conducting the study and their family members, ages 2 to 30 years old. Individuals were recruited using e-mails and flyers. These materials included basic information about the study (e.g., study title, who was conducting the study, how to obtain more information). Interested individuals contacted the study investigators to enroll in the study.

*Consent Process.* Both studies followed the Code of Federal Regulations that stipulates all federally funded research must convey the following information as part of the consent process: 1. why the research is being conducted, 2. what participants will be asked to do, 3. whether participation is voluntary, 4. who is conducting the research, 5. who the participant can contact for information, 6. whether there are health risks associated with participating, 7. what benefits may result from participation, and 8. to what extent participant confidentiality will be maintained [14]. This information was conveyed in a written consent form (parents of child VUCS participants were given a parental consent form). To further standardize these forms, each was reviewed and approved by the same Institutional Review Board (IRB). Potential participants were instructed to read the form, ask questions pertaining to the material, and to sign the form if they were willing to participate.

The NAHP consent form contained 1,301 words. The VUCS consent forms for adult participants and for parents of child participants contained 994 and 1,088 words, respectively (Table 1). The Flesch-Kincaid Reading levels [15] (FKR) for the NAHP and VUCS consent forms were below an eighth grade reading level (Table 1). The FKR for the comprehension assessment tools was below a sixth grade reading level (Table 1).

*Assessment of Consent Comprehension*. Information needed to obtain informed consent requires a three-step process [16,17]. First, a potential study participant must receive information; second, they must comprehend the received information; and third, they must choose whether to use what they comprehended to aid in making a decision. Thus to determine whether a participant has made an informed consent researchers might measure the participant’s comprehension and the use of the comprehended information to make their informed decision. However, comprehension is difficult to measure and therefore a standard proxy for comprehension is to measure an individual’s ability to recognize and recall information they have received [6,7,9,16,18–20]. Recognition addresses the participant’s ability to recognize content provided in the consent form and is measured using multiple choice and yes/no/unsure formatted questions. Open-ended questions are used to assess the subject’s ability to recall information described in the consent process. If a person is not able to recognize or recall conveyed information, they will subsequently not be able to comprehend or use that information to make an informed decision regarding their study participation.

Our comprehension assessment tool measured each participant’s comprehension of six required consent form elements (Table 2). We used recognition to measure comprehension of information on voluntary participation (3 questions), study methods (7 questions), risk of participation (5 questions for the NAHP and 4 for the VUCS), and confidentiality (1 question). Each question was phrased as a statement requiring either a yes/no/unsure response. The following elements were measured using recall: benefits of participation (1 question), and study objectives (1 question). Recall questions were open-ended questions.

All questions addressed information described in the two primary environmental health studies’ consent forms. In an effort to make the NAHP and VUCS comprehension assessment tools comparable, similar questions and wording were used. Recognition questions on voluntary participation and confidentiality were identical in both studies (e.g., “I may choose to stop participating at any time?” and “My identifying information will be used when presenting the study results to the public?”), as were recall questions concerning benefits of participation (e.g., “In 1 sentence, describe what if any is the immediate benefit of participating in this study.”) and study objectives (e.g., “In 1–2 short sentences, describe why this study is being done.”). The questions on study methodology (what the participant would be asked to do) and risks of participation addressed study-specific information and therefore the wording varied between the two studies. The risks associated with participating in the NAHP included exposure to radiation from the x-ray and other minimal risks (e.g., dizziness, light headed). Risks for VUCS participants included temporary urine discoloration and the identification of glucose or other compounds that are not normally found in urine.

Although the VUCS included both adult participants and parents of child participants, the comprehension assessment tools contained virtually the same questions; the difference was in the object of the sentence. For example, the voluntary participation question for adult participants read, “I choose freely to join in this study?” while the question for parents read, “I choose freely to let my child join in this study?” The wording and clarity of the questions were reviewed and edited by the investigator’s IRB. The comprehension assessment tools for the NAHP and VUCS are available from the authors upon request.

To measure overall consent comprehension, each of the six required elements contributed one point to a total comprehension score of 6 points. Points accumulated for correct scores only. The responses “do not know” and “unsure” were classified as incorrect. Interviewers were instructed to probe each participant about questions left unanswered. This was done to assess whether participants purposefully refused to answer the question or if they left the question blank because they did not know the answer. As there were no individuals who stated they purposefully refused to answer a question, all unanswered questions were classified as incorrect responses. For the elements with more than one question, each sub-part contributed an equal proportion to the total score of 1 point. For example, there were three questions on voluntary participation. Thus, each of the three questions contributed 0.33 points to the total score of 1 point. For the open-ended recall questions, researchers developed a list of correct responses. Participant responses were then independently reviewed and scored by two researchers. Discrepancies in scores among the two researchers were then reviewed and discussed before a final correct or incorrect score was designated.

*Administering the Comprehension Assessment Tool.* For each study, interviewers were trained to ensure that all participants received the same information during the consent process. Specifically, interviewers met with participants and reviewed standardized communication points (e.g., study title, who was conducting the study). The interviewer asked each participant to read the consent form, to ask questions if necessary, and to sign the consent form if they wanted to participate. Without the participant’s knowledge, interviewers recorded the total length of time each participant took to review the consent form. To assess whether specific questions or parts of the consent form were unclear, the interviewer documented all questions asked by the participant.

An interviewer asked all NAHP participants who reviewed and signed the NAHP consent form, on one of five recruitment days, to participate in the consent study by answering a few questions about the NAHP consent form they had just signed. All VUCS adult participants and parents of minors who did not assist in the development of the VUCS study protocol or the consent material were asked to participate in the consent study by answering a few questions about the VUCS consent form. If the participant agreed, he or she was asked to answer the questions on the comprehension assessment tool to the best of their ability. After completing the comprehension assessment tool the interviewer discussed the correct answers with the participant and verbally reconfirmed their willingness to participate.

*Demographic Data*. Each of the primary environmental health studies collected demographic data on the primary study participant’s gender, age, and race/ethnicity. These data were used to analyze potential differences in observed consent comprehension. Shortly after the VUCS study began, approval was received to collect the same demographic data from consenting VUCS parents of child participants

Since the VUCS target population included adults who worked for a public health agency, familiarity with conducting human health studies and developing consent forms could influence the level of comprehension assessed. To control for this potential bias we asked each VUCS consent study participant whether they developed or reviewed consent forms, or study protocols as part of their work.

*Data Analyses.* Data were entered into Epi Info version 3.3.2 (Centers for Disease Control and Prevention, Atlanta, GA). All survey responses were double entered to ensure data quality. The data were then analyzed using SAS version 9.1.3 (SAS Institute, Inc., Cary, NC). We assessed whether total comprehension was associated with gender, race and ethnicity (Non Hispanic White *vs.* Other) using the Wilcoxon rank-sum test. To determine whether age group was associated with comprehension, the Kruskal-Wallis test was used. Lastly, the Spearman rank correlation coefficient was used to assess the relationship between each participant’s total comprehension score and the amount of time they spent reviewing the consent form.

## 3. Results

All NAHP and VUCS participants asked to participate in the consent comprehension study agreed to participate. This included 10 NAHP participants and 63 VUCS participants for a total of 73 people. The VUCS participants consisted of 21 adult participants and 42 parents of child participants. With one exception, all comprehension assessment tools were self-administered; for one individual the VUCS interviewer verbally read the comprehension assessment questions to the participant.

*Demographics.* The NAHP participants differed from the VUCS participants on gender and age (Table 3). Eighty percent (n = 8) of the NAHP participants were male compared to 42% (n = 63) of the VUCS participants. Similarly 80% (n = 8) of the NAHP participants were between the ages of 51 and 76 compared to only 10% (n = 5) of the VUCS participants. Age was not collected on 11 of the VUCS parents. Forty percent (n = 25) of VUCS participants stated that they developed or reviewed study protocols or consent forms. More parents of child participants developed or reviewed these materials compared to adult VUCS participants (Table 3).

*Comprehension*. Only three people had perfect comprehension scores. Comprehension scores ranged from 1.91 to 6.00 with an aggregate mean of 4.66 (95% CI: 4.44, 4.88). Overall mean comprehension was statistically different by study. The NAHP participants scored on average 3.72 (95% CI: 2.88, 4.56) compared to 4.81 (95% CI: 4.60, 5.02) for VUCS participants (Table 4). The VUCS participants scored significantly higher on comprehension of issues pertaining to voluntary participation, study methodology and confidentiality (Table 4).

The comprehension of potential study-related risks were similar among both NAHP (Mean = 0.62; 95% CI: 0.38, 0.86) and VUCS (Mean = 0.59; 95% CI: 0.51, 0.67) participants (Table 4). Of the five questions pertaining to study-related risks, 60% (n = 6) of the NAHP participants were unaware of two or more risks. Similarly, 52% (n = 33) of the VUCS participants were unaware of two or more risks. Eight percent (n = 5) of VUCS participants answered all four risk related questions incorrectly.

The differences in comprehension observed across the two studies remained the same after restricting the analyses to only those participants who did not develop or review study protocols or consent forms as part of their regular work. After reviewing the consent form, 27 people asked a question about the study before they signed the form. The majority of questions asked concerned appointment scheduling or whether normal daily routines could be followed while participating in the study (e.g., can a multi-vitamin be taken while participating). There was no statistical association between overall comprehension and having asked a question before signing the consent form.

The amount of time a participant spent reviewing the consent form was recorded for 65 participants. The mean reviewing time was 2.06 minutes (range: 0.00–11.00 minutes). On average NAHP participants reviewed the form for a slightly longer period of time, 4.49 minutes (range: 0.00–11.00 minutes) compared to 1.71 minutes (range: 0.03–4.73 minutes) for VUCS participants. On average, high school students read between 214 and 250 words per minute [21]. Using standard reading rates for comparison, the majority of our participants spent insufficient time reviewing the consent form. Standard reading rates suggest NAHP participants should have spent at least 5 minutes reading the consent form; 4 minutes for VUCS participants.

There was no relationship between total comprehension score and time spent reviewing the consent form. There were weak correlations between a participant’s total comprehension score and the demographic factors gender and age; however, after stratifying by study (NAHP *vs.* VUCS) these correlations were no longer evident (data not shown).

## 4. Discussion

The study-related risks are likely the most important information a researcher must convey in the consent form. However, our participants scored low on study-related risks. It is possible that study participants would have been more inclined to consider the risks had they been of greater magnitude (more than minimal risk). However, previous research suggests otherwise; participants in placebo-controlled clinical trials and those scheduled for invasive medical procedures also have limited comprehension of consent form material [6,7]. Thus, it is reasonable to assume that people who consent to environmental health studies with more than minimal risk, such as some biomonitoring and genetic research studies, may also not fully comprehend the associated risks.

Given our participants were the least aware of the study-related risks, this poses the question, “how do we relay consent material in a way that study participants receive and comprehend what we wish to convey.” Some researchers suggest using bulleted information and plain language [22]. One of the most popular methods is reducing the reading level of the form to one that is appropriate for the target population [23–25]. In our study, a low reading level did not ensure comprehension. Others have advocated that the consent process be recorded so that researchers could identify problems and suggest corrective measures [26]. The use of multimedia has also been considered; multimedia may include asking potential participants to view a short video or to partake in an interactive computer program [27–29]. However, Flory and Emanuel’s review found verbal communication on study-related benefits and risks was the best method to improve comprehension when compared to other multimedia approaches [30].

Researchers also need to consider why individuals choose to participate. Do they participate because of a sense of enlightened self-interest in reducing scientific uncertainty? One study suggests that altruism is a factor [31]. Some people will participate regardless of whether they understand the risks or benefits; people participate because they desire to help others. Others advocate that some subjects participate because of a therapeutic misconception in which participants think researchers want to promote the participant’s individual health [32,33]. Given individuals participate for different reasons, understanding the predominant reasons will aid researchers in developing consent forms more suited for their target population.

*Limitations.* To our knowledge a validated tool for assessing comprehension among environmental health study participants does not exist. Therefore, we developed and used non-validated tools. Although it was not within the scope of this study to test the reliability and validity of the tools, the questions were reviewed and approved by scientists with expertise in human subjects research as well as the author’s IRB. It is also important to mention our study included a small sample of individuals from two distinct target populations. As the majority of the participants worked for a large public health agency, our participants may have had a greater awareness and knowledge of public health studies and practices. Alternatively, participants may have been less inclined to consider the material they were given as part of the consent material. While it is difficult to know how generalizable our results might be of the greater general population, our participants are likely to represent a broader cross section of the population compared to those who have been studied previously (the elderly, substance abusers, the mentally challenged, and participants in clinical trials) [6–13] and our results are similar to larger studies that found comprehension of traditional consent form material to be low [6,7,34–36]. In addition, NAHP and VUCS study participants differed on demographics and overall mean comprehension scores, thus we report study specific as well as the aggregate data. Another limitation is that we were restricted to demographic data collected by the primary environmental health studies. This prohibited us from collecting additional data such as each participant’s educational attainment or reading level.

*Conclusion.* In environmental health, researchers have successfully improved community studies by seeking community involvement in the design stage. Specifically, community members have successfully aided researchers in determining how to measure exposure (e.g., which exposures to look for, where to site environmental monitors), and have aided in increasing an individual’s willingness to support and participate in research studies [37–40]. We propose that preliminary discussions with members of the target community also include dialogue on reasons why people may participate and how to best convey consent material such as study related risks. These preliminary discussions with community members may shed light on how to improve consent comprehension among individuals who are asked to participant in environmental public health studies.

## Figures and Tables

**Table 1.** Flesch-Kincaid Reading Level^*^ for Informed Consent Forms and Comprehension Assessment Tools Used by Each Study.

**Study**	**Word Count**	**Informed Consent Form**	**Comprehension Assessment Tool**
**National Asbestos Health Program**			
All participants	1,301	7.9	5.4
**Variation in Urinary Creatinine Study**			
Adult participants	994	7.2	4.9
Parents of child participants	1,088	7.1	5.1

* Indicates the approximate U.S. grade level of the written text based on the average number of syllables per word and the average number of words per sentence.

**Table 2.** Overview on How Consent Comprehension Was Measured.

**Required Consent Form Element**	**Comprehension Assessment Method**	**Number of Questions Asked**
Voluntary participation	Recognition	3
Study methodology^a^	Recognition	7
Potential risks to the study participant	Recognition	5/4^b^
Confidentially	Recognition	1
Benefits of participation	Recall	1
Study objectives	Recall	1

**Total Number of Elements**		18/17^b^

a Study methodology included questions on what participants would be asked to do, who was conducting the study, and who the participant should contact for additional information;

b The NAHP included one extra question compared to the VUCS.

**Table 3.** Participant Characteristics by Study.

	**NAHP**		**VUCS**						**TOTAL**	

	**All Participants (n = 10)**		**All Participants (n = 63)**		**Adult Participants (n = 21)**		**Parents of Child Participants (n = 42)**		**(N = 73)**	

	N	%	N	%	N	%	N	%	N	%
**Gender**										
Male	8	80	22	42	11	52	11	35	30	48
Female	2	20	30	58	10	48	20	65	32	52
Missing	0		11		0		11		1	

**Age Group**										
20–30	1	10	20	38	20	95	0	0	21	34
31–50	1	10	27	52	1	5	26	84	28	45
51–76	8	80	5	10	0	0	5	16	13	21
Missing	0		11		0		11		11	

**Race\Ethnicity**										
Non Hispanic White	6	67	39	78	16	84	23	74	45	76
Other	3	33	11	22	3	16	8	26	14	24
Missing	1		13		2		11		14	

**Develops or reviews study protocols or consent forms**										
Yes	-	-	25	40	5	24	20	49	-	-
No	-	-	37	60	16	76	21	51	-	-
Missing			1		0		1			

% = Percent; N = Number; NAHP = National Asbestos Health Program; VUCS = Variation in Urinary Creatinine Study.

**Table 4.** Mean Comprehension Scores for Consent Form Elements by Study.

**Consent Form Element**	**Total Possible Score**	**Overall (N = 73)**	**NAHP (n = 10)**	**VUCS (n = 63)**	**P-value^a^**
**Overall Comprehension**	6	4.66	3.72	4.81	0.01
Voluntary participation	1	0.98	0.90	0.99	<0.01
Study methodology	1	0.84	0.70	0.86	0.01
Potential risks to the study participant	1	0.60	0.62	0.59	0.86
Confidentiality	1	0.85	0.20	0.95	<0.01
Benefits of participating	1	0.77	0.70	0.78	0.59
Study objectives	1	0.63	0.60	0.63	0.83

NAHP = National Asbestos Health Program; VUCS = Variation in Urinary Creatinine Study;

a Calculated 95% p-value represents statistical difference between the NAHP and VUCS studies.
